# Robust Electrodes for Flexible Energy Storage Devices Based on Bimetallic Encapsulated Core–Multishell Structures

**DOI:** 10.1002/advs.202100911

**Published:** 2021-05-29

**Authors:** Yan‐Fei Li, Shuyang Ye, Yan‐Hong Shi, Jian Lin, Yi‐Han Song, Yang Su, Xing‐Long Wu, Jing‐Ping Zhang, Hai‐Ming Xie, Zhong‐Min Su, Hai‐Zhu Sun, Dwight S. Seferos

**Affiliations:** ^1^ College of Chemistry National & Local United Engineering Laboratory for Power Batteries Northeast Normal University 5268, Renmin Street Changchun 130024 P. R. China; ^2^ Department of Chemistry University of Toronto 80 St. George Street Toronto Ontario M5S 3H6 Canada

**Keywords:** bimetallic sulfides, core–multishell structure, flexible electrode, lithium/sodium‐ion batteries

## Abstract

Developing flexible electrodes with high active materials loading and excellent mechanical stability is of importance to flexible electronics, yet remains challenging. Herein, robust flexible electrodes with an encapsulated core‐multishell structure are developed via a spraying‐hydrothermal process. The multilayer electrode possesses an architecture of substrate/reduced graphene oxide (rGO)/bimetallic complex/rGO/bimetallic complex/rGO from the inside to the outside, where the cellulosic fibers serve as the substrate, namely, the core; and the multiple layers of rGO and bimetallic complex, are used as active materials, namely, the shells. The inner two rGO interlayers function as the cement that chemically bind to two adjacent layers, while the two outer rGO layers encapsulate the inside structure effectively protecting the electrode from materials detachment or electrolyte corrosion. The electrodes with a unique core‐multishell structure exhibit excellent cycle stability and exceptional temperature tolerance (−25 to 40 °C) for lithium and sodium storage. A combination of experimental and theoretical investigations are carried out to gain insights into the synergetic effects of cobalt‐molybdenum‐sulfide (CMS) materials (the bimetallic complex), which will provide guidance for future exploration of bimetallic sulfides. This strategy is further demonstrated in other substrates, showing general applicability and great potential in the development of flexible energy storage devices.

## Graphical abstract

The encapsulated core–multishell structure with an architecture of substrate/reduced graphene oxide (rGO)/bimetallic complex/rGO/bimetallic complex/rGO is used to fabricate robust and high energy density electrodes for lithium/sodium storage, with a high proportion of active material (20 wt%) and mechanical strength. The underlying synergistic effect of bimetal ions is revealed via experimental investigations and theoretical calculations.

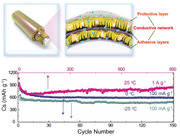

## 1 Introduction

The rising demand for flexible electronics has been driving the pursuit of flexible batteries with high bending‐ or folding tolerance.^[^ ^1^, ^2^, ^3^, ^4^, ^5^, ^6^, ^7^, ^8^, ^9^, ^10^, ^11^, ^12^ ^]^ To fabricate flexible electrodes, one of the most promising strategies is to grow the active materials in situ onto various self‐standing and flexible carbon‐ or metal‐based substrates, such as carbon cloth, graphene, nickel foam, and etc.^[^ ^13^, ^14^, ^15^, ^16^, ^17^, ^18^, ^19^ ^]^ However, the current flexible electrode configurations are still facing some limitations. The proportion of active materials is usually too low to afford high energy density, which in turn limits the practical applications to energy storage devices. This is due to either the relatively high weight ratio of the substrates or the insufficient sites for growing the active materials. Additionally, the active materials attachment and monolithic electrode structure are difficult to maintain upon deformation. Therefore, the gravimetric/areal specific capacity (*C*_s_/*C*_a_) and cycle stability of current flexible electrodes are still far from satisfactory, especially at high power outputs and extreme temperatures.

In order to improve the weight ratio of active materials, many efforts have been devoted to increasing the specific surface area of substrates. For example, Tong and co‐workers reported a porous carbon cloth substrate by an etching technique.^[^ ^20^ ^]^ However, the common etching process is detrimental to the mechanical strength of the substrates. Additionally, the overall improvement by increasing the surface area is limited by the number of layers of active materials. Intuitively, depositing multilayer active materials on a flexible substrate is very useful to fabricate high‐loading flexible electrodes.^[^ ^16^, ^21^ ^]^ For instance, Zhang and co‐workers reported a multilayer structure, where commercial level mass loading was achieved by stacking two layers of VS_2_ materials on a stainless steel mesh.^[^ ^21^ ^]^ Nevertheless, the adhesion between the active materials and the substrate turns out to be weak and directly stacking layers of active materials leads to poor interlayer contacts. The combined effects result in increased internal resistance and decreased utilization of the active materials. Finally, this loosely stacked multilayer structure easily leads to active materials detachment, particularly when a sharp bending event occurs.^[^ ^22^, ^23^ ^]^ Therefore, constructing a multilayer flexible electrode with improved interlayer contacts and superior stability, especially at various bending states, remains as a pivotal challenge.

With the aforementioned issues in mind, one should consider the following aspects when designing multilayer flexible electrodes with a high proportion of active material. First, a lightweight and flexible substrate should be adopted to reduce the weight of non‐active components. Second, an integrated and conductive network should be constructed to improve the intralayer and interlayer electron transfer and the utilization of active materials. An ideal conductive network should also be able to bridge the layers reinforcing the interlayer adhesion and stabilizing the flexible electrode, in particular at deformation states.^[^ ^24^, ^25^ ^]^ Conventionally, carbon‐based materials are constructed on the surface of active materials to improve the stability or conductivity. However, complicated heating treatment is often involved and sometimes results in destruction of active materials and substrates. Especially, it is difficult to construct an independent conductive layer that simultaneously works for another active materials layer growth in multilayer flexible electrodes. Therefore, a new strategy and structure are essential for multilayer flexible electrodes.

**Scheme 1:**
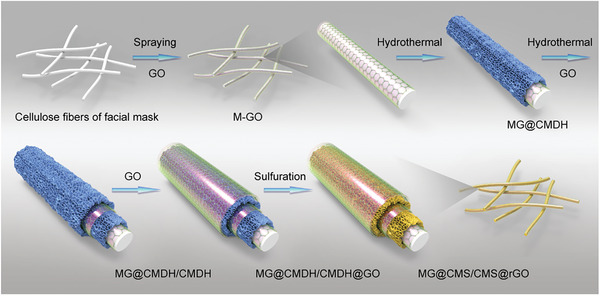
Schematic illustration of the preparation of encapsulated core–multishell MG@CMS/CMS@rGO composite.

Herein, a cost‐effective and scalable flexible electrode with encapsulated core–multishell structure is described. As shown in **Scheme** **1**, the electrode possesses an architecture of substrate/reduced graphene oxide (rGO)/bimetallic complex/rGO/bimetallic complex/rGO from inside to outside. A common, commercially available face‐mask is used as the flexible substrate, and cobalt–molybdenum–sulfide (CMS) possessing rich electrochemistry and high electron conductivity is used as the redox‐active complex.^[^ ^26^, ^27^ ^]^ The multiple layers of rGO and CMS are considered as the active materials. A total of three separated layers of rGO form a conductive network. The innermost layer of rGO functions as an adhesion layer between the substrate and the active materials while the outmost layer functions as an encapsulation layer for the internal structure. The middle layer has both functions. Benefiting from the advantageous design and architecture of the electrode (denoted as MG@CMS/CMS@rGO), high capacity and superior cycle stability were achieved, especially at low operating temperatures, when employed in lithium‐ion batteries (LIBs) and sodium‐ion batteries (SIBs). Moreover, a series of measurements and density functional theory (DFT) calculations reveal the electrochemical mechanisms and electron transfer process in CMS.

## 2 Results and Discussion

**Figure 1:**
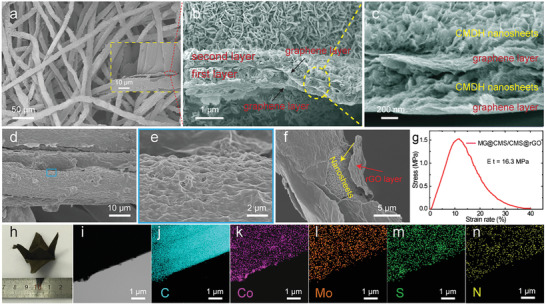
a–c) SEM of MG@CMDH/CMDH precursor and the exposed inner structure (inset a). b,c) The corresponding zoomed‐in view of the inner structure, revealing the multilayer shell structure. d,e) SEM of MG@CMS/CMS@rGO fiber, indicating that the internal multilayer structure is tightly encapsulated by the outmost rGO layer. f) After intentionally splitting the outermost rGO, the CMS nanosheets are observed, keeping the morphology of their precursor CMDH. g) Stress–strain curve of MG@CMS/CMS@rGO, showing the excellent mechanical strength. h) Digital photograph of MG@CMS/CMS@rGO folded into a crane without damage. i) TEM image and j–n) EDX elements mapping of MG@CMS/CMS@rGO.

The in‐depth morphology characterization of the encapsulated core–multishell MG@CMS/CMS@rGO is illustrated in **Figure** **1**. A common face mask composed of micron‐sized cellulose fibers (Figure S1, Supporting Information) exhibits high flexibility and good hydrophilicity (Figure S2, Supporting Information), which makes it an excellent candidate for a flexible substrate. The mask was first coated by a nanolayer of wrinkled graphene oxide (denoted as M‐GO, Figure S3, Supporting Information). Graphene oxide (GO) is rich in negatively charged oxygen‐containing functional groups, which provides abundant binding sites for growing the active materials.^[^ ^28^ ^]^ Co–Mo double hydroxide (CMDH) precursors were then grown on the surface of M‐GO via electrostatic adsorption and hydrothermal assembly (denoted as MG@CMDH, Figure S4, Supporting Information). Interestingly, at a feed ratio of Co to Mo ≈7:1, a uniform and dense nanosheet array was formed (Figure 1a–c, Figure S5, Supporting Information), which determines the morphology after the sulfidation reaction. During the hydrothermal reaction, GO is simultaneously reduced to rGO leading to enhanced electronic conductivity.^[^ ^15^ ^]^ In contrast, no CMDH nanosheets were observed (Figure S6, Supporting Information) when the hydrothermal reaction was carried out on an undecorated mask under the same conditions. This is likely due to the absence of growth sites for CMDH, and this provides further evidence for the necessity of the coated GO layer.

With only one layer of CMS nanosheets, the mass loading and proportion are approximately 0.3 mg cm^−2^ and 10%, respectively, which is low compared to the requirement for high energy density. In order to increase the amount of active materials, a second layer of CMDH nanosheets was grown following encapsulating the first CMDH layer with a GO nanolayer (denoted as MG@CMDH/CMDH, Figure 1a–c). From the lateral exposed region of the coating layer (inset of Figure 1a), two CMDH layers with similar thickness of ≈0.7 µm are observed. The multilayer shell structure with rGO/CMDH layer/rGO/CMDH layer is further confirmed by magnifying the region in yellow dashed line (Figure 1b). It is observed that CMDH layers are tightly attached to the rGO layers in turn furnishing a sturdy multilayer structure and a favorable conductive network. In order to protect the integrated electrode, another layer of GO was coated to encapsulate the whole multilayer structure (Figure 1d,e). After intentionally cracking the outermost rGO using an ultrasonic bath, the CMS nanosheets are observed, which is consistent with the morphology of CMDH nanosheets (Figure 1f). After sulfidation, the final MG@CMS/CMS@rGO electrode has a mass loading of active materials significantly increased to about 0.8 mg cm^−2^ and the proportion active materials increases to approximately 20 wt.%.

Surprisingly, without coating an rGO layer between the two layers of CMS, poor morphology and severe detachment of CMS were observed (Figure S7, Supporting Information), leading to a much lower mass loading of approximately 0.4 mg cm^−2^. Electrodes without the outmost rGO encapsulation were also prepared. Here, the multilayer structure was destroyed during the course of sulfidation (Figure S8, Supporting Information). These combined results suggest how critical the rGO interlayer is for the structural stability and integrity of these composite electrode materials. The exceptional flexibility and stability of the final MG@CMS/CMS@rGO electrode are illustrated in Figure 1h, where no damage is observed after sharply folding the electrode. Moreover, the MG@CMS/CMS@rGO has excellent mechanical strength with Young's modulus of 16.3 MPa (Figure 1g), which is better than most reported carbon‐based flexible electrodes.^[^ ^16^, ^29^ ^]^ Worth mentioning, the final electrode inherits the shape and size of the mask substrate (Figure S9, Supporting Information), which shows the applicability for preparing large‐area flexible electrodes.

**Figure 2:**
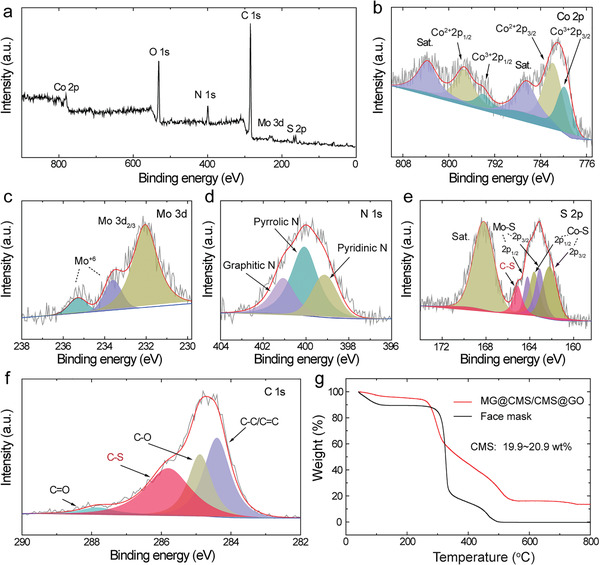
a) XPS survey spectra and b–f) high‐resolution XPS spectra of Co 2p, Mo 3d, N 1s, S 2p, and C 1s of MG@CMS/CMS@rGO, demonstrating the presence of CMS. g) TGA curves of MG@CMS/CMS@rGO and face mask showing a high CMS proportion of approximately 19.9–20.9 wt% in the electrode.

Elemental mapping confirms the presence of C, Co, Mo, and S in the MG@CMS/CMS @rGO (Figure 1i–m). X‐ray diffraction (XRD) suggests that the CMS nanosheets are amorphous (Figure S10, Supporting Information). The stoichiometry of CMS is estimated to be Co_4‐5.6_MoS_10.76_, as determined by a combination of X‐ray photoelectron spectroscopy (XPS, **Figure** **2a**) and inductively coupled plasma mass spectroscopy (ICP‐MS, Table S1, Supporting Information) analysis. High‐resolution XPS spectra were recorded to determine the chemical environments of the various elements in the electrode. Two pairs of peaks corresponding to Co^3+^ and Co^2+^ as well as two satellite peaks are observed by fitting the Co 2p XPS spectrum (Figure 2b).^[^ ^18^ ^]^ In CMS, the Mo exhibits an oxidation state of +4 with a small fraction being oxidized to Mo^6+^ in air (see Mo 3d XPS spectrum in Figure 2c).^[^ ^30^ ^]^ In the S 2p spectrum, an obvious shoulder is observed at 165.1 eV along with the peaks corresponding to the Mo—S and Co—S bonds (Figure 2e). This shoulder is assigned to C—S bond, which is further confirmed by the C 1s spectrum (Figure 2f).^[^ ^31^ ^]^ The formation of C—S bonds is likely due to the S doping the rGO during sulfidation. O and N heteroatoms are also detected in the XPS. The O heteroatoms are mostly from the rGO layers. The N heteroatoms with three differentiable subsets of pyridinic N, pyrrolic N, and graphitic N likely stem from the hexamethylenetetramine used in the hydrothermal processes (Figure 2d).^[^ ^32^ ^]^ Both the elemental mapping (Figure 1n) and elemental analysis (Table S2, Supporting Information) confirm the presence of N heteroatoms (about 1.23 wt% in MG@CMS/CMS@rGO). S and N codoping the rGO improves the electron transport kinetics in the electrochemical process, which further benefits the performance of the MG@CMS/CMS@rGO electrode.^[^ ^33^ ^]^

CMS accounts for 19.9–20.9% of the mass of the final electrode, which is calculated from thermogravimetric analysis (TGA; Figure 2g; see the calculation details in the experimental section). This agrees well with the weight analysis during the electrode fabrication (Table S3, Supporting Information). Remarkably, owing to the lightweight flexible substrate (≈3 mg) and the multilayer structure layout, the weight ratio (≈20%) of the active material to the flexible electrode is effectively improved and much higher than literature values where most report less than 10% and only a few reach 16% (Table S4, Supporting Information).

**Figure 3:**
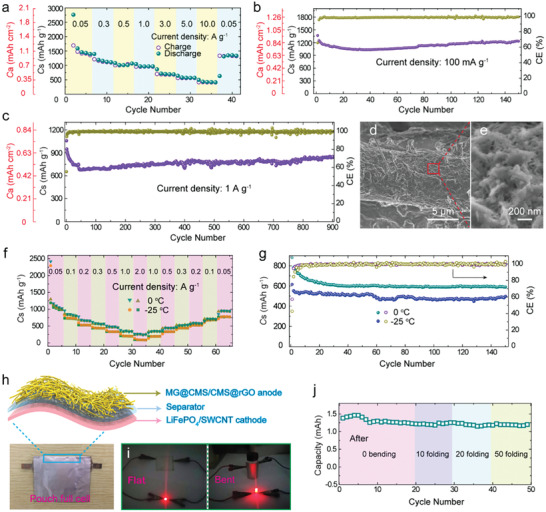
Electrochemical properties of MG@CMS/CMS@rGO. a) Rate performances at various current densities. Cycling performances at b) 100 mA g^−1^ and c) 1 A g^−1^ at room temperature (≈25 °C). d,e) SEM images of MG@CMS/CMS@rGO after 900 cycles at 1 A g^−1^. f) Rate performances, g) cycling performances at 100 mA g^−1^ at 0 °C and ‐25 °C, respectively. h) Schematic illustration and digital photo of MG@CMS/CMS@rGO//LiFePO_4_/SWCNT pouch full cell. i) The pouch full cell drives the LED lights under flat and bent states, respectively. j) Cycling performance of the full cell at different folding numbers.

To evaluate the performance of the electrode, lithium‐ion batteries were fabricated. The face mask substrate barely contributes to the overall capacity (Figure S11, Supporting Information). The discharge–charge profiles of MG@CMS/CMS@rGO exhibit multiple charge/discharge plateaus, implying the rich electrochemical reactions (Figure S12, Supporting Information). The MG@CMS/CMS@rGO shows high reversible capacities of 1415, 1130, 1006, 958, 680, 557, and 410 mAh g^−1^ at 0.05, 0.3, 0.5, 1.0, 3.0, 5.0, and 10.0 A g^−1^, respectively (**Figure** **3a**). A reversible capacity of 1348 mAh g^−1^ is retained after 40 cycles when the current density is reduced to 0.05 A g^−1^, indicating outstanding electrochemical reversibility and electrode stability. It is worthwhile to mention that the lightweight MG@CMS/CMS@rGO electrode also possesses a remarkable *C*_a_ of 1 mAh cm^−2^ at 0.05 A g^−1^, resulting from the high loading of active materials. More importantly, the excellent reversible *C*_s_ of 1232 mAh g^−1^ (corresponding to *C*_a_ of 0.86 mAh cm^−2^) is maintained after 150 cycles at 0.1 A g^−1^ with an excellent capacity retention of 90% and an average Coulombic efficiency (CE) over 99% (Figure 3b). When the current density is increased to 1 A g^−1^, the MG@CMS/CMS@rGO electrode retains a charge *C*_s_ of 850 mAh g^−1^ (corresponding *C*_a_ of 0.59 mAh cm^−2^) after 900 cycles exhibiting a low capacity degradation of 0.021% per cycle (Figure 3c).

To investigate the impact of the number of CMS layers on performance, the electrodes with one (MG@CMS@rGO) and two layers of active materials were fabricated and compared. Even though their *C*_s_ are comparable, the *C*_a_ of MG@CMS@rGO is about one third of that of MG@CMS/CMS@rGO (Figure S13, Supporting Information). In the electrodes absent of the middle and outmost rGO layers, the *C*_s_ drops significantly to 400 and 25 mAh g^−1^ (corresponding to *C*_a_ of 0.14 and 0.01 mAh cm^−2^) at 0.1 and 1 A g^−1^, respectively (Figures S14 and S15, Supporting Information). This is due to the poor morphology and the detachment of CMS active materials during cycling.

The electrode morphology does not change after 900 cycles when examined by scanning electron microscopy, demonstrating the stability of fabricated electrodes (Figure 3d,e). The excellent mechanical strength and flexibility of the electrode are maintained after cycling tests (Figure S16, Supporting Information). Comparatively, without the outmost rGO layer, the electrode suffers from severe performance loss after 500 cycles (Figure S17, Supporting Information). To investigate the mechanism of the excellent rate performance of MG@CMS/CMS@rGO, the pseudocapacitive contributions for Li storage were calculated from CV curves at varied scan rates (Figure S18, Supporting Information). The proportion of pseudocapacitive charge storage increase with increasing scan rates (22%, 29%, 34%, 43% and 56% at the scan rates of 0.1, 0.3, 0.5, 1.0 and 2.0 mV s^−1^), which is beneficial for high‐rate performance.^[^ ^10^ ^]^

Electrochemical impedance spectra (EIS) measurements reveal that the ohmic resistance (*R*_s_) value after 50 cycles (3.228 Ω) is comparable to the pristine state (3.113 Ω), indicating the formation of a stable solid electrode/electrolyte interface upon cycling. Meanwhile, the charge transfer resistance (*R*_ct_) decreases from 162.5 to 57.4 Ω, which is ascribed to sufficient electrolyte infiltration and the increased exposure of electrochemical active surface area after charge‐discharge cycles (Figure S19 and Table S5, Supporting Information).^[^ ^34^ ^]^ Such excellent electrochemical property greatly benefits from the unique electrode structure. The vertically aligned nanosheet array with interconnected pores in CMS layers is responsible for the fast ion transfer. The two inner rGO welding layers strongly bind to the CMS layers preventing active materials from detaching even under huge volume change and provide a conductive network to enhance the utilization of active materials. The encapsulating rGO layers fully protect the electrode from the corrosion of electrolyte and keep the integrity of the flexible electrode.

The MG@CMS/CMS@rGO electrode also shows superior properties at high/low temperatures (Figure S20, Supporting Information, Figure 3f,g). As shown in Figure 3f, the rate capacities at ‐25 °C are comparable to those at 0 °C. When the current density reaches 500 mA g^−1^, a high capacity retention of 76% is achieved at ‐25 °C compared to the capacity at 0 °C. Moreover, after 150 cycles, the cells at 0 and ‐25 °C retain the outstanding reversible capacities of 588.6 and 497 mAh g^−1^ at 100 mA g^−1^ with a capacity retention of 67% and 81%, respectively (Figure 3g). The excellent rate capacities and cycle stability at low temperature are attributed to the stable structure and fast electron/ion transfer, as explained above. Strikingly, the cycle stability of the MG@CMS/CMS@rGO electrode is fairly competitive among the reported ternary metal oxides/sulfides, as summarized in Figure S21 and Table S6 (Supporting Information) (only the MG@CMS/CMS@rGO electrode in this work possesses excellent cycle stabilities at temperatures ranging from ‐25 °C to 25 °C).

In order to evaluate the potential to apply the MG@CMS/CMS@rGO electrodes to flexible batteries, a flexible cathode composed of LiFePO_4_ and single‐walled carbon nanotube (SWCNT) network was prepared (Figures S22 and S23, Supporting Information). The full MG@CMS/CMS@rGO//LiFePO_4_/SWCNT pouch cell with dimensions of 1.5 × 2 cm^2^ was assembled as illustrated in Figure 3h. Based on the discharge plateau of cathode and charge plateaus of the anode, the full cell exhibits two obvious discharge plateaus at approximately 2 and 1 V, thus agreeing with the charge–discharge curves (Figure S24, Supporting Information). As a result of the excellent mechanical flexibility and stability, there are not any obvious fluctuations in the cell performances at different folding cycles. Even after folding for 50 times, the pouch full cell delivers a desirable capacity of 1.2 mAh at 0.5 mA, which is equivalent to a gravimetric energy density of 127 Wh kg^−1^ (Figure 3j). Furthermore, the flexible pouch cell is able to power commercially available LEDs upon severe deformation, showing no observable difference to the intact batteries (Figure 3i).

**Figure 4:**
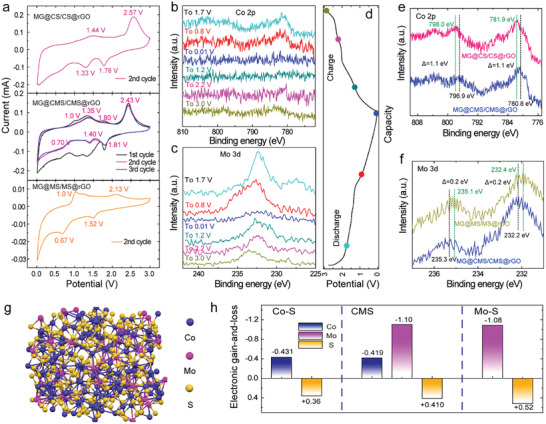
a) CV curves of MG@CMS/CMS@rGO, MG@CS/CS@rGO, and MG@MS/MS@rGO. The ex situ XPS spectra of MG@CMS/CMS@rGO b) Co 2p and c) Mo 3d at various first charge–discharge states indicated in the corresponding d) charge–discharge profiles in the first cycle. e) Co 2p spectra of MG@CMS/CMS@rGO and MG@CS/CS@rGO. f) Mo 3d spectra of MG@CMS/CMS@rGO and MG@MS/MS@rGO. g) Optimized cluster structure of CMS. h) The electronic gain‐and‐loss of CMS, Co‐S and Mo‐S derived from DFT calculations (+ and – represent electron gain and loss, respectively).

Even though the bimetallic sulfides have been widely explored as anode materials, the underlying lithium storage mechanism remains ambiguous. In this respect, cyclic voltammograms (CV) were conducted to better understand the reaction process of MG@CMS/CMS@rGO (**Figure** **4a**). The electrodes with similar structures but utilizing only cobalt sulfide or molybdenum sulfide as active materials are used as references (denoted as MG@CS/CS@rGO and MG@MS/MS@rGO, respectively, see the corresponding cycle stabilities in Figure S25, Supporting Information). In the first cathodic scan, four reduction peaks centered at ≈1.80, ≈1.32, ≈1.05, and ≈0.65 V are clearly observed for MG@CMS/CMS@rGO. The peak at 1.05 V is absent in the subsequent cycles, which is attributed to the inevitable formation of solid electrolyte interphase (SEI) film. The peaks at ≈1.80 and ≈1.32 V correspond to the insertion of Li^+^ into the CMS (Equation 1), while the peak at ≈0.65 V corresponds to the formation of metallic Co and Mo (Equation 2). Combining the CV curves of MG@CS/CS@rGO and MG@MS/MS@rGO, two pairs of anodic peaks at ≈1.00, ≈1.80 V and ≈1.35, ≈2.43 V are observed, corresponding to the conversion of Mo and Co into molybdenum sulfides and cobalt sulfides, respectively (Equations (3) and (4)).

(1) $$Co_{4-5.6}Mo_{1}S_{10.76}+xLi^{+}+xe^{-}\to Li_{x}Co_{4-5.6}Mo_{1}S_{10.76}$$

(2) $$\begin{array}{ccc} & & Li_{x}Co_{4-5.6}Mo_{1}S_{10.76}+\left(21.52-X\right)Li^{+}\\ & & \;+\;\left(21.52-X\right)e^{-}\to 4-5.6\mathrm{Co}+\mathrm{Mo}+10.76Li_{2}S \end{array}$$

(3) $$\mathrm{Mo}+xLi_{2}S↔\mathrm{Mo}S_{x}+2xLi^{+}+2xe^{-}$$

(4) $$\mathrm{Co}+xLi_{2}S↔\mathrm{Co}S_{x}+2xLi^{+}+2xe^{-}$$

In the subsequent cathodic scans, the peaks at 1.81 and 1.40 V are due to the reduction of cobalt sulfides into metallic Co while the peaks at 1.40 and 0.70 V are ascribed to the reduction of molybdenum sulfides into metallic Mo. Note that, the reduction peaks in close proximity at 1.33 V (in MG@CS/CS@rGO) and 1.52 V (in MG@MS/MS@rGO) fuse into one peak for MG@CMS/CMS@rGO located at 1.40 V.

Ex situ XPS of MG@CMS/CMS@rGO at various charge/discharge states in the first cycle further confirms the above‐mentioned electrochemical reaction processes (Figure 4b–d). When discharging to 1.7 and 0.8 V, the peak intensity of Co and Mo remains unchanged, indicating the occurrence of insertion reaction. While after the full discharge, the peaks of Co disappear, indicating the complete conversion of the Co ions into ultrasmall Co nanocrystals. Owing to the coverage of Li_2_S and SEI film, the metallic Co is hardly detected in the XPS measurements. On the contrary, Mo peaks with lower intensity after discharging to 0.01 V, suggesting the incomplete conversion of Mo ions. The different behaviors imply that Co ions may be more reactive than Mo ions. The Mo peaks strengthen after charging to 1.2 and 2.2 V while the Co profile has no clear change, demonstrating the exclusive recovery of Mo ions. After finally charging to 3.0 V, the Co peaks fully recover. Moreover, the peaks of the Co and Mo XPS clearly are positively shifted, indicating the formation of molybdenum sulfides and cobalt sulfide rather than CMS after the first cycle, in agreement with the CV results.

Interestingly, compared to MG@CS/CS@rGO and MG@MS/MS@rGO, the polarization of MG@CMS/CMS@rGO decreases to a large extent, suggesting enhanced reaction kinetics. Due to the similarity in structures of the three electrodes, the enhanced performance of MG@CMS/CMS@rGO possibly originates from the synergistic effect between Co and Mo. However, the underlying origin for such a synergistic effect has not been revealed in detail. Herein, the density functional theory (DFT) calculations were employed to understand this origin. After optimizing the cluster structure of the CMS (Figure 4g), Co–S (Figure S26, Supporting Information) and Mo–S (Figure S27, Supporting Information), the electronic gain‐and‐loss number of every element is acquired, as displayed in Figure 4h. Evidently, Co in CMS loses less electron density (‐0.419) than Co–S (‐0.431). Inversely, the Mo in CMS loses more electron density (‐1.1) compared to Mo‐S (‐1.08). This indicates the occurrence of electron transfer from Mo to Co in the bimetallic CMS, resulting in the increased electron density in Co, which emerges as the electrochemically active centers. This explains why Co tends to be more reactive as observed in the ex situ XPS. Furthermore, the XPS spectra of the three electrodes are compared to illustrate the electron distribution change on the Co and Mo ions (Figure 4e,f). Compared with the MG@CS/CS@rGO, the peaks of Co 2p in MG@CMS/CMS@rGO shift negatively, indicating the increased electron density of Co. In reverse, Mo 3d peaks (Figure 4f) in MG@CMS/CMS@rGO shift positively relative to the MG@MS/MS@rGO owing to more electron loss. Taken together, these results demonstrate that the inherent synergistic effects of the two metal ions in the bimetallic sulfides are derived from electron transfer that forms electrochemically active centers and this is responsible for the improved electrochemical reaction kinetics.

**Figure 5:**
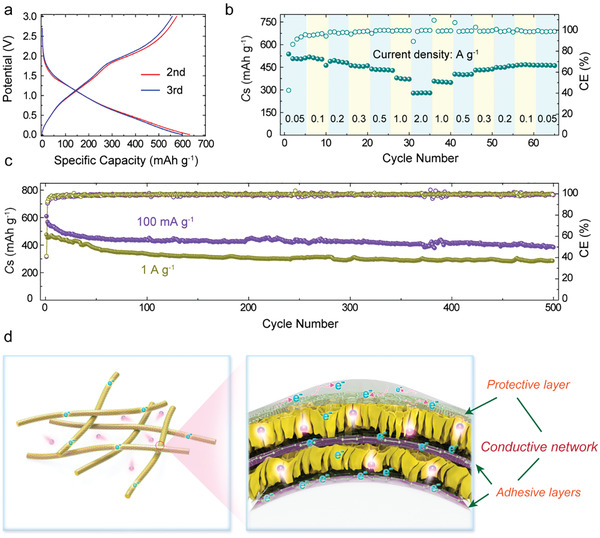
The electrochemical performance of MG@CMS/CMS@rGO as the anode of SIBs, a) Discharge–charge profiles for the second and third cycles. b) Rate capability at various current densities at room temperature. c) Cycling performance at a current density of 100 mA g^−1^ and 1 A g^−1^. d) Schematic illustration the advantages of the encapsulated core–multishell structure in electrochemical reaction.

In addition to LIBs, this MG@CMS/CMS@rGO electrode was also tested in SIBs. The first cathodic scan of the CV profile displays three peaks at ≈1.04 V, ≈0.72 V, and ≈0.01 V, respectively (Figure S28a, Supporting Information), which decrease by 0.3 V relative to the peaks in LIBs. This indicates that the MG@CMS/CMS@rGO electrode in SIBs experiences similar conversion reactions to the LIBs. The charge‐discharge plateaus are coincident with CV curves, and the overlapping from the second cycle suggests excellent electrode stability (Figure S28b, Supporting Information and **Figure** **5a**). The MG@CMS/CMS@rGO delivers excellent cycle stability (Figure 5c) with reversible capacities of 385 and 286 mAh g^−1^ after 500 cycles at 0.1 and 1.0 A g^−1^, respectively. Moreover, the MG@CMS/CMS@rGO exhibits outstanding rate performances (Figure 5b). Even at 2.0 A g^−1^, a reversible capacity of 208 mAh g^−1^ is still achieved. Moreover, the MG@CMS/CMS@rGO electrode shows favorable low‐temperature performance as a SIB anode. When the current density was increased from 50 mA g^−1^ to 500 mA g^−1^, the considerable capacities of 200.7 and 190.2 mAh g^−1^ are retained at 0 °C and ‐15 °C, respectively (Figure S29, Supporting Information). The outstanding electrochemical performance is mainly ascribed to the stable structure and superior electron/ion transfer kinetics of the multilayer structure as schematically illustrated in Figure 5d. Furthermore, this strategy is further demonstrated to be applicable to other hydrophilic substrates, such as the commercially available handkerchief paper and cloth (Figures S30 and S31 and Table S3, Supporting Information). Therefore, there is considerable potential to extend the scope of this method to prepare flexible electrodes from other substrates and active materials.

## 3 Conclusion

In summary, flexible CMS electrodes with the encapsulated core–multishell structure were successfully prepared with a high weight ratio of 20% of active materials. The interconnected nanosheet networks of the two layers of active materials provide abundant space for quick electrolyte infiltration and ion diffusion. In addition to a conductive network, the rGO layers serve as chemical binders holding all the layers together and as an encapsulant protecting the electrode from destruction and corrosion. Consequently, the MG@CMS/CMS@rGO electrode exhibits excellent cycle stability and capacities in LIBs and SIBs especially at low temperatures. CV and ex situ XPS disclose that the CMS is transformed into binary‐phase molybdenum sulfides and cobalt sulfides after the first cycle, and the reversible conversion reactions occur in the subsequent cycles. In addition, the electron transfer from Mo to Co, which is supported by the DFT calculations and XPS results, leads to improved activity of Co and thus enhanced electrochemical reaction kinetics. The in‐depth understanding of the synergistic effect of two metal ions provides future guidance for the exploration of bimetallic sulfides. This work provides an effective, scalable, and universal strategy to prepare durable multilayer flexible electrodes, showing appreciable potential in flexible batteries applications.

## Conflict of Interest

The authors declare no conflict of interest.

## Supporting information

Supporting Information

## Data Availability

The data that support the findings of this study are available from the corresponding author upon reasonable request.
