# Supplementary material for: Robust Electrodes for Flexible Energy Storage Devices Based on Bimetallic Encapsulated Core–Multishell Structures
Source: Adv Sci (Weinh). 2021 May 29;8(14):2100911. doi: 10.1002/advs.202100911 (PMC8292853; doi:10.1002/advs.202100911)
Supplement: Supplementary file 1 — Supporting Information [file ADVS-8-2100911-s001.pdf]

WILEY-VCH

© 2021 Wiley-VCH GmbH

ADVANCED SCIENCE
Open Access

Supporting Information

for *Adv. Sci.,* DOI: 10.1002/advs.202100911

Robust Eletrodes for Flexible Energy Storage Devices based on Bimetallic Encapsulated Core-Multishell Structures

Yan-Fei Li, Shuyang Ye,Yan-Hong Shi, Jian Lin, Yi-Han Song, Yang Su, Xing-Long Wu, Jing-Ping Zhang, Hai-Ming Xie, Zhong-Min Su, Hai-Zhu Sun, and Dwight S. Seferos*

Supporting Information

Robust Eletrodes for Flexible Energy Storage Devices based on Bimetallic Encapsulated Core-Multishell Structures

Yan-Fei Li, Shuyang Ye ,Yan-Hong Shi, Jian Lin, Yi-Han Song, Yang Su, Xing-Long Wu, Jing-Ping Zhang, Hai-Ming Xie, Zhong-Min Su, Hai-Zhu Sun, Dwight S. Seferos*

Y. F. Li, Dr. Y. H. Shi, J. Lin, Y. H. Song, Y. Su, Prof. X. L. Wu, Prof. J. P. Zhang, Prof. H. M. Xie, Prof. Z. M. Su, Prof. H. Z. Sun
College of Chemistry, National & Local United Engineering Laboratory for Power Batteries
Northeast Normal University
5268, Renmin Street, Changchun, 130024, P. R. China
E-mail: sunhz335@nenu.edu.cn

Dr. S. Y. Ye, Prof. D. S. Seferos
Department of Chemistry, University of Toronto
80 St. George Street, Toronto, Ontario M5S 3H6, Canada

Keywords: lithium/sodium-ion batteries, flexible electrode, bimetallic sulfides, core-multishell structure

Experimental Section

Materials: Hexamethylenetetramine ($C_6H_{12}N_4$), cobalt nitrate hexahydrate ($Co(NO_3)_2 \cdot 6H_2O$), ammonium molybdate ($(NH_4)_2MoO_4$), hydrazine hydrate (H_6N_2O) and thioacetamide (CH_3CSNH_2) were all analytical grade. Graphene oxide (GO) and commercially used face mask paper (composed with cellulose fibers) were also used in preparation processes.

Synthesis of M-GO: the face masks were washed sequentially with acetone, ethanol and deionized water several times to remove unwanted impurities. Then, dried in a vacuum oven at 60 °C for 12 h. Graphene oxide (GO) was prepared as previously reported.[1] GO was dispersed in deionized water to obtain the GO solution (2 mg mL^{-1}) after vigorous stirring and ultrasonication. The face masks were first completely moistened by GO solution and then dried at 60 °C for 12 h. Meanwhile, the face mask changed from white to gray.

Synthesis of MG@CMDH/CMDH@GO precursor: $C_6H_{12}N_4$ (7 mmol), $Co(NO_3)_2 \cdot 6H_2O$ (3 mmol) and $(NH_4)_2MoO_4$ (0.42 mmol) were added into deionized water (70 mL) to form the light pink solution. After vigorous stirring for 12 h, 20 μL of H_6N_2O was injected into the solution. Then, the solution and M-GO substrate were transferred into a 100 mL autoclave and heated at 160 °C for 24 h. The product after first hydrothermal reaction was washed with deionized water and dried in a vacuum oven at 60 °C for 12 h to obtain MG@CMDH. After that, MG@CMDH was second completely wetted by GO solution and dried in a vacuum oven at 60 °C for 12 h, and then underwent the second hydrothermal reaction that was same as the first. After dried in vacuum oven, the products were third wetted by GO solution to get the final protective barrier. The MG@CMDH/CMDH@GO precursor was obtained after dehydrated in a vacuum oven.

Synthesis of MG@CMS/CMS@rGO: CH_3CSNH_2 (62.5 mg) was added into 62.5 mL deionized water under continuous stirring for 5 min. Then, the solution was transferred into a 100 mL autoclave in the presence of the MG@CMDH/CMDH@GO precursor. The autoclave

was then heated at 120 °C for 3 h. After naturally cooling to room tempreature, the black product MG@CMS/CMS@rGO was rinsed several times with deionized water and dried in vacuum oven. The mass loading of active materials was 0.6~0.8 mg cm^{-2} determined from the difference in weight of face masks before and after reaction, which includes CMS nanosheets and rGO.

Synthesis of MG@CS/CS@rGO and MG@MS/MS@rGO: for comparison, MG@CS/CS@rGO and MG@MS/MS@rGO were obtained through the same conditions as MG@CMS/CMS@rGO except for just adding $Co(NO_3)_2{\cdot}6H_2O$ (3 mmol) in MG@CS/CS@rGO and $(NH_4)_2MoO_4$ (3 mmol) in MG@MS/MS@rGO.

Synthesis of MG@CMS/CMS@rGO-14 and MG@CMS/CMS@rGO-3.6: in order to investigate the Co and Mo synergistic effect on morphology, MG@CMS/CMS@rGO-14 and MG@CMS/CMS@rGO-3.6 were obtained through changing the $(NH_4)_2MoO_4$ contents to 0.21 mmol and 0.84 mmol, respectively. The experiments were under the same way as MG@CMS/CMS@rGO.

Synthesis of $LiFePO_4$/SWCNT cathode: single-walled carbon nanotube (SWCNT) were added into N,N-dimethylformamide for 2h ultrasonic treatment to obtain homogeneous SWCNT suspension with a concentration of 0.5 mg mL^{-1}. After that, 35 mg of commercial $LiFePO_4$ was added into the SWCNT suspension (30 mL), followed by other 1h ultrasonication to form $LiFePO_4$/SWCNT ink. Finally, $LiFePO_4$/SWCNT films with the planar density of about 6.4 mg cm^{-2} were obtained by a vacuum filtration method. The mass loading of $LiFePO_4$ in $LiFePO_4$/SWCNT electrode is ~ 4.5 mg cm^{-2}.

The assembly of electrode: the MG@CMS/CMS@GO were cut into disks with 1.2 cm diameter as the working electrode. For lithium-ion batteries (LIBs), pure lithium foil was used as the counter electrode and a 1.0 M $LiPF_6$ in 1:1 v/v ethylene carbonate (EC)/dimethyl carbonate (DMC) as the electrolyte. For sodium-ion batteries (SIBs), pure sodium foil was used as the counter electrode and 1.0 M $NaClO_4$ in 1:1 v/v propylene carbonate and ethylene

carbonate as the electrolyte. The 2032 coin cells were assembled in an Ar-filled glovebox (S-Universal 2440/750, from MIKROUNA) with the concentrations of moisture and oxygen below 0.1 ppm.

The electrochemical measurement: for the electrochemical measurements, the cyclic voltammogram (CV) measurements were carried out ranging from 0.01 to 3.00 V at a scan rate of 0.1 mV s^{-1}. The galvanostatic charge-discharge testing was conducted on the battery chargers (Land, CT2001A). The electrochemical impedance spectroscopy (EIS) measurements were performed in half cells at 5 mV AC amplitude over the frequency range of 0.1 MHz-0.01 Hz under the open-circuit condition by electrochemical workstation (Princeton, P4000). The low temperature property of the cells was evaluated on battery testing systems. During the whole testing period, the connecting line of the instrument as well as the fabricated CR2032 coin cells were both in a refrigerator.

The assembly of pouch full cell: the pouch full cell included Al-plastic file, Ni battrey tabs, MG@CMS/CMS@rGO anode and $LiFePO_4$/SWCNT cathode, diaphragm, and electrolyte. Adopting hot-seal peaking machine packages the pouch cell in Ar-filled glovebox. Before assembling the full-cells, the MG@CMS/CMS@rGO anode was chemical prelithiation for 5 cycles at the current density of 100 mA g^{-1}. The electrochemical performance of the pouch full cell was evaluated in the potential window of 0.7–3.7 V.

Characterizations: the morphology and structure of the obtained materials were observed by scanning electron microscopy (SEM, XL 30 ESEM-FEG, FEI Company) and transmission electron microscopy (TEM, JEM-2010F). X-ray diffractometer (XRD) patterns were recorded on a Rigaku SmartLab X-ray diffractometer. X-ray photoelectron spectroscopy (XPS) was performed with an energy step size of 0.1 eV using Al Ka radiation.

Calculations of TGA:

TGA measurement was carried out at air atmosphere in 10°C min^{-1} heating rate. TGA curve of face mask showed a large reduction of weight from 100% to 0% starting from

~30 °C up until 500 °C, indicating a complete decomposition. The weight loss from ~30 °C to ~530 ^{o}C in TGA curve of MG@CMS/CMS@rGO was due to the decomposition of face mask and rGO, as well as the bimetallic sulfides into metal oxides by oxidation. Afterward, the weight was kept nearly constant up until 800 °C. The stoichiometry of CMS was estimated to be $Co_{4\sim5.6}Mo_1S_{10.76}$. The content of CMS in MG@CMS/CMS@rGO was about 19.9~20.9%, which was calculated according to the following equation:

$$m_{Co_{4\sim5.6}Mo_1S_{10.76}} = \frac{\Delta m}{M_{Co_{4\sim5.6}Mo_1O_{10.76}}} \times M_{Co_{4\sim5.6}Mo_1S_{10.76}} \times 100\% \qquad (1)$$

Where M, Δm and $m_{Co_{4\sim5.6}Mo_1S_{10.76}}$ are the molar mass, the content of $Co_{4\sim5.6}Mo_1O_{10.76}$ (14.8%) and $Co_{4\sim5.6}Mo_1S_{10.76}$, respectively.

DFT calculation:

The first-principle calculations were performed by using Vienna ab initio simulation package with the Perdew-Burke-Ernzerhof-type generalized gradient approximation for the exchange-correlation functional and the projector augmented wave method. The structure of these amorphous materials were built in a supercell with more than 200 atoms and simulated with a born-oppenheimer molecular dynamics at the NVT ensemble with 600 K in 6 ps. After the MDs, all the atoms were relaxed until the residual force was less than 0.05 eV/Å. A 2 × 2 × 2 grid of k points and a plane-wave cutoff energy of 400 eV were used for the self-consistent calculations and bader charge analysis.

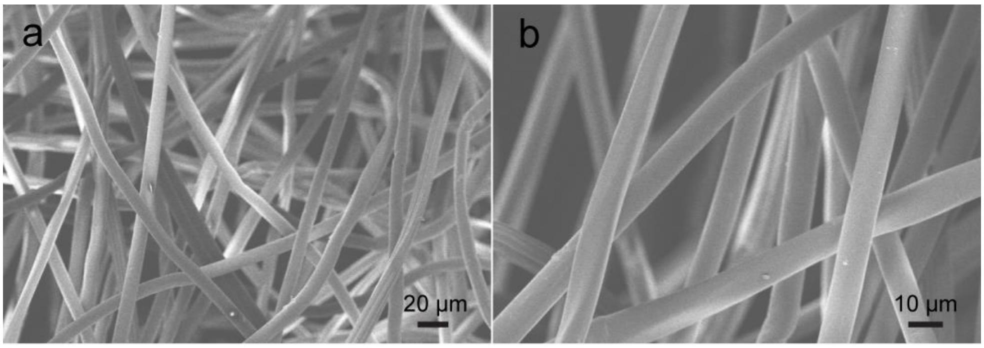

Figure S1 a-b) SEM images of the pristine face mask with crossed fiber, exhibiting the smooth surface.

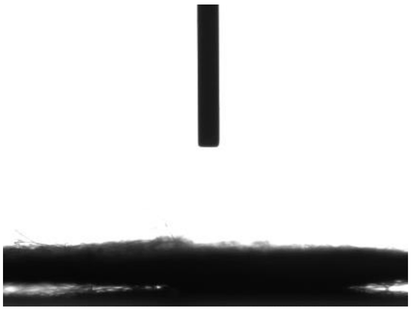

Figure S2 Water contact angles for face mask, showing that the water was completely absored immediately and hence a good hydrophilicity.

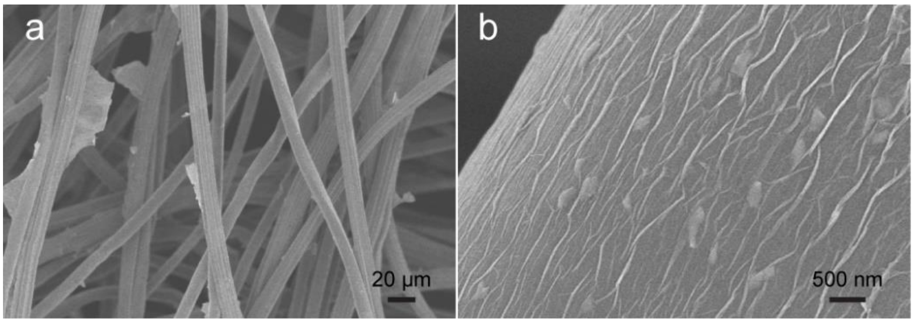

Figure S3 a, b) SEM images of M-GO, the mask fibers were firmly coated by GO nanolayer with the wrinkled surface.

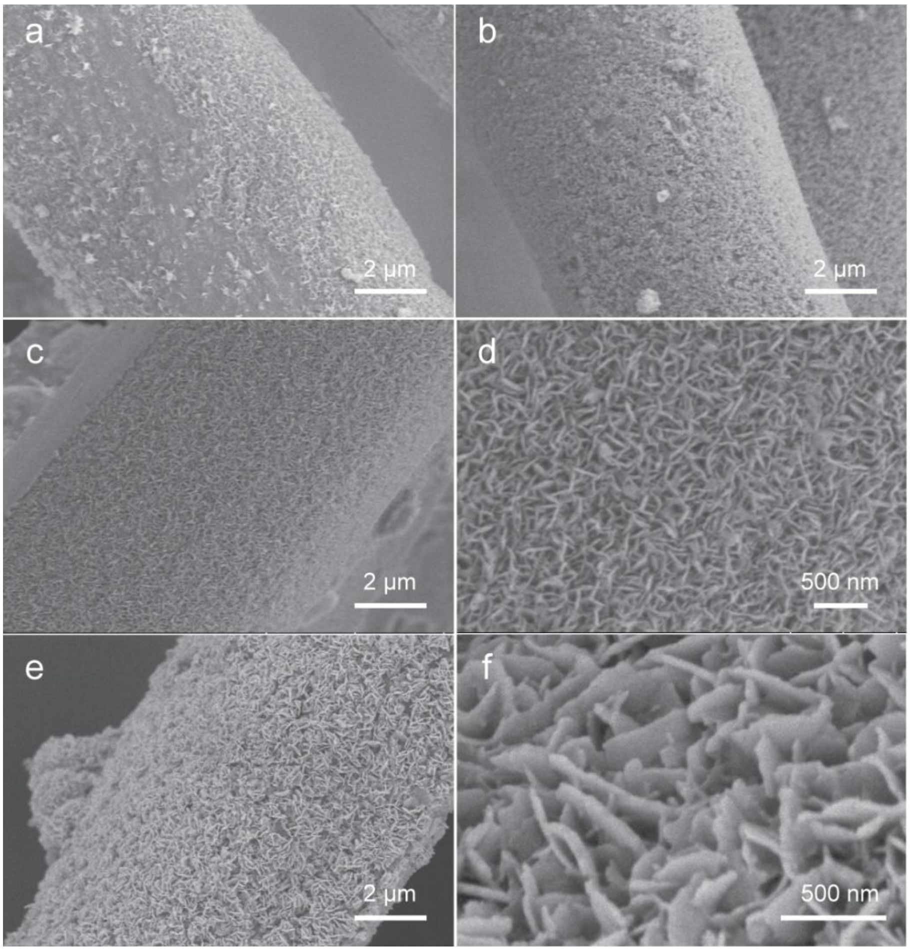

Figure S4 The process of MG@CMDH precursor self-assembled into parallelly aligned nanosheets on M-GO substrate. SEM images of products at the reaction times a) 4 h, b) 8 h, c, d) 12 h, e, f) 24 h. The CMDH precursor nucleated on the surface of M-GO and then grew into nanosheet array due to the electronegative GO surface.

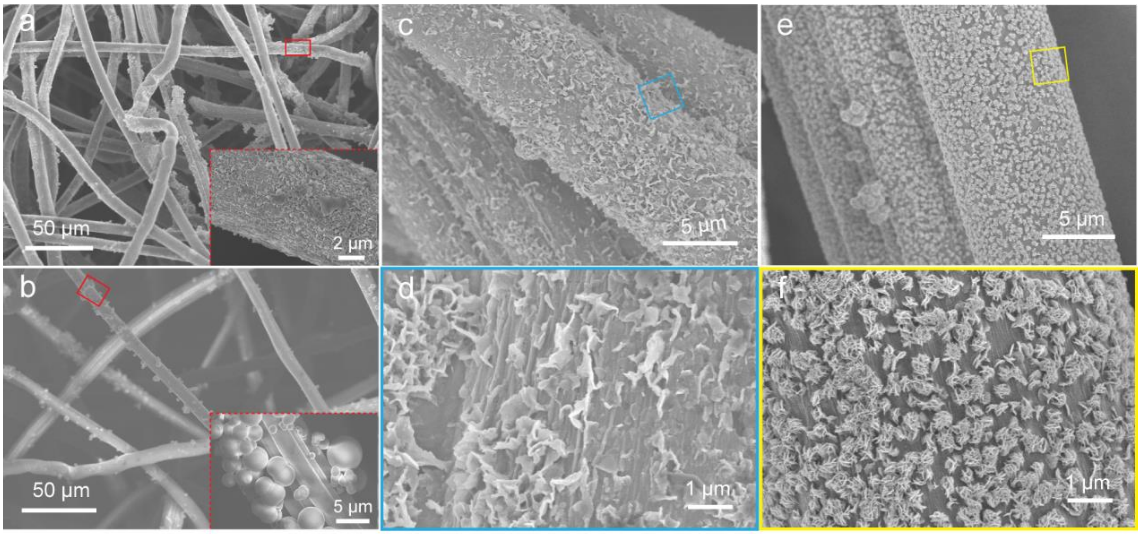

Figure S5 The morphology control base on the ratio of Co salt to Mo salt. SEM images of a) MG@Co hydroxide, b) MG@Mo hydroxide, c, d) MG@CMDH-14 (the ratio of $Co(NO_3)_2 \cdot 6H_2O$ to $(NH_4)_2MoO_4$ is 14), and e, f) MG@CMDH-3.6 (the ratio of $Co(NO_3)_2 \cdot 6H_2O$ to $(NH_4)_2MoO_4$ is 3.6).

Figure S5 demonstrates that the morphology of CMDH precursors is dependent on the ratio of elements Mo to Co. Only when the Co salt is added (MG@Co hydroxide), the nanosheets are inclined to grow parallelly on the nanofibers, but the nanosheets are irregular and dispersed unevenly (Figure S5a). When the Mo salt is added (MG@Mo hydroxide), the sparse and different-sized nanospheres are obtained (Figure S5b). Furthermore, when the ratio of Co salt to Mo salt equals to 14 (MG@CMDH-14) (Figure S5c, d), the nanosheets become denser compered with MG@Co hydroxide but still discretely distribute on the nanofibers relative to MG@CMDH with the Co salt to Mo salt equals to 7, demonstrating the presence of Mo improves the interaction between the nanosheets and M-GO substrate. When the ratio of Co salt to Mo salt equals to 3.6 (MG@CMDH-3.6), the trend toward spherification for Mo increases, and the nanosheets are assembled into flower-shape nanospheres. Conclusively, the ratio of metals has a significant effect on the morphology construction for bimetallic sulfides. Only the ratio of Co salt to Mo salt equals to 7, the uniform and dense nanosheet array is

achieved to form a flat surface, leading to more electrochemical active sites and quicker ion diffusion.

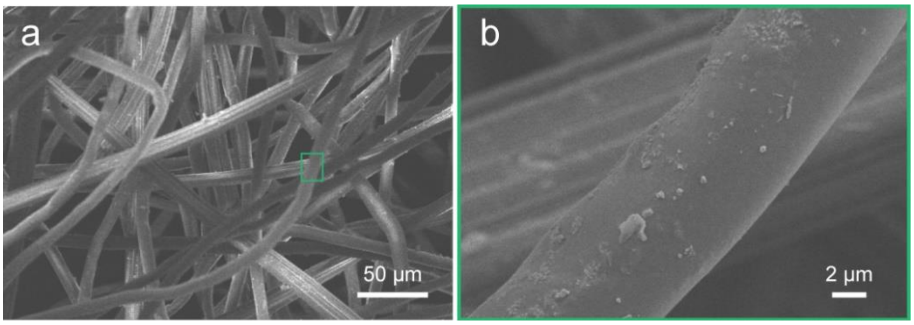

Figure S6 a, b) the SEM images of pure face mask as substrate for growth of CMDH precursors via a hydrothermal progress. Almost no CMDH nanosheets were observed due to the absence of growth sites for CMDH, suggesting the necessity of the coated GO layer.

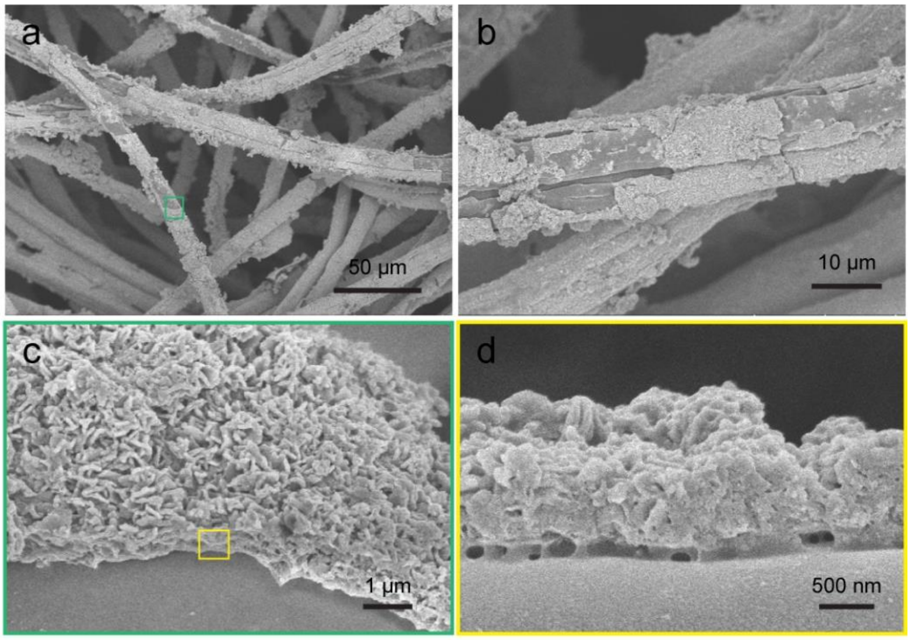

Figure S7 The SEM images of MG@CMS/CMS@rGO without middle and outermost rGO layers, showing that the CMS active materials with poor nanosheets structure fall off from face mask substrate seriously (a, b), and the multilayer structure is unobvious (c, d).

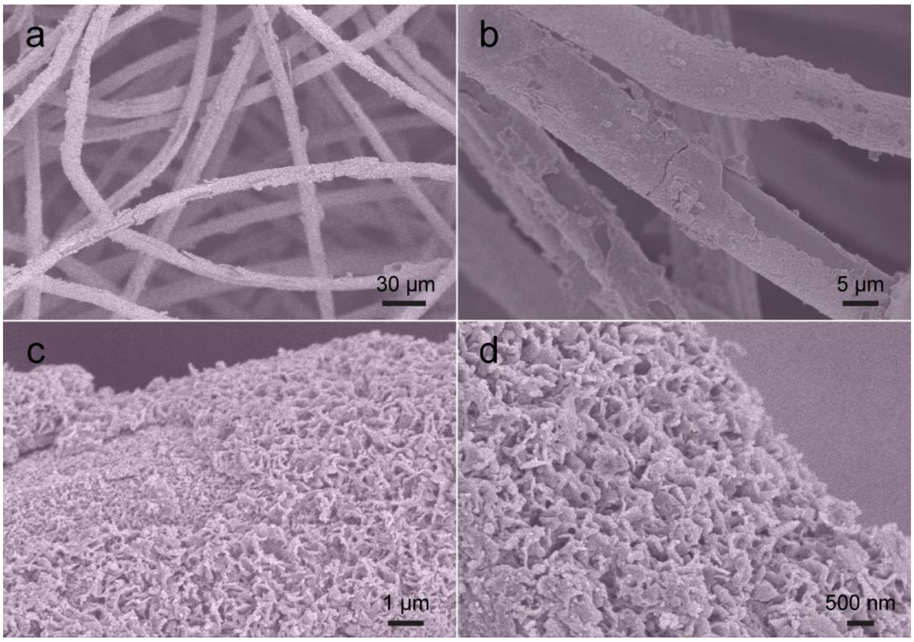

Figure S8 SEM images of MG@CMS/CMS@rGO without the protection of outermost rGO nanolayer at verious magnification. It is clear that the active materials partly detach from mask fibers (a, b) and the multilayer structure suffers severe damages (c, d).

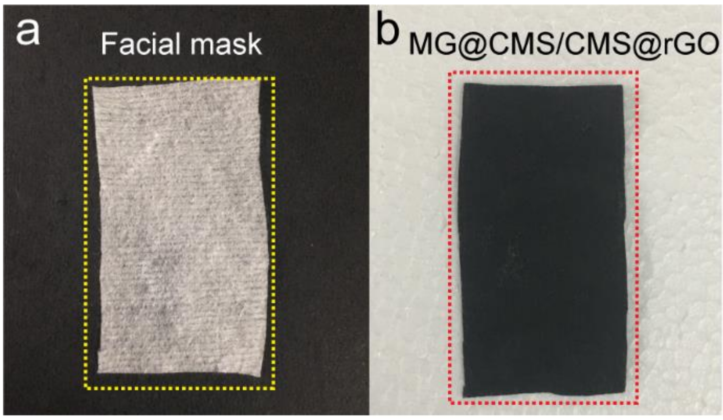

Figure S9 The photograph of face mask (a) before and (b) after reaction. There is no reduction for the size, showing the feasibility to prepare the large-size flexible electrode for practical application.

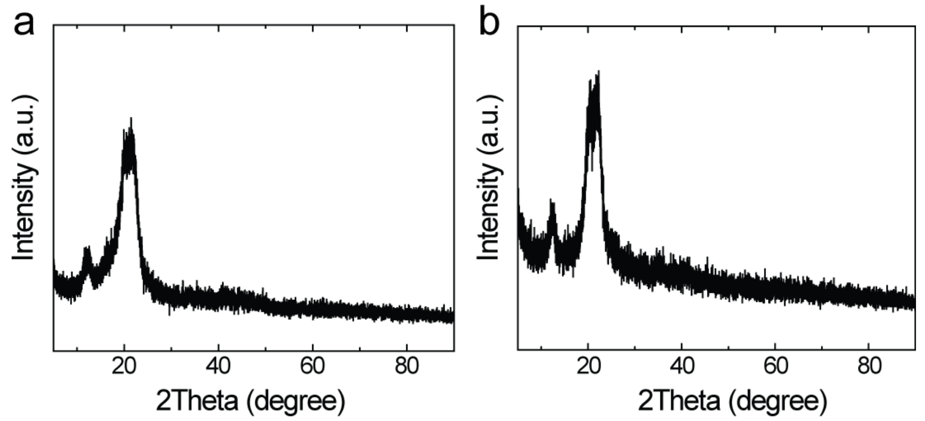

Figure S10 The XRD patterns of a) pristine face mask, b) MG@CMS/CMS@rGO. Except for face mask peaks, no peaks were detected in MG@CMS/CMS@rGO, indicating CMS nanosheets are amorphous.

Table S1 The ICP results of molar ratios of Co to Mo in MG@CMS/CMS@rGO at the feed ration of Co to Mo equal to 7.

Sample	The content of Co	The content of Mo	ICP (Co:Mo)
MG@CMS/CMS@rGO	2.8	0.5	5.6

Table S2 Elemental analysis of face mask and MG@CMS/CMS@rGO. The content of N in MG@CMS/CMS@rGO is much higher than face mask (0.04 wt.%), which originated from the hexamethylenetetramine during the hydrothermal process.

Sample	C (wt.%)	N (wt.%)
Face mask	40.035	0.04
MG@CMS/CMS@rGO	35.88	1.23

Table S3 The proportion of active materials in different electrodes with 12 mm diameter.

Skeleton	Mass (mg)	Active materials (mg)	Proportion of Active materials
Face mask	~ 3	0.7 ~ 0.9	17 ~ 21%
Handkerchief Paper	~ 1.7	~ 0.37	18%
Cloth	~ 12.5	~ 2	14%

Table S4 Comparision the advantages of core-multishell structure for MG@CMS/CMS@rGO in this work with other free-standing materials.

Products	Skeleton	Flexible and binder-free	The mass of electrode (mg cm^{-2})	The proportion of active materials in electrode	Ref.
**MG@CMS/ CMS@rGO**	**Cellulose fiber**	**Yes**	**3.8**	**~ 20.0%**	**This work**
SnNA	Cu foil	No	> 8.68	< 7.8%	[2]
CoP@PPy NWs/CP	Carbon cloth	Yes	> 11.2	< 10.7%	[3]
VONW-CF	Carbon cloth	Yes	> 11.8	< 15.3%	[4]
NCNW-CF	Carbon cloth	Yes	> 11.2	< 10.7%	[4]
N-PSi@C	Stainless steel foil	No	23.85	3.6%	[5]
VS_4-CC@ VS-3	Carbon cloth	Yes	16.43	8.6%	[6]
Ni-Mo-S NS	Carbon cloth	Yes	> 11.8	< 15.3%	[7]
Ni-Fe-S NS	Carbon cloth	Yes	> 11.9	< 16.0%	[7]
ZCO@CFT	Carbon cloth	Yes	> 11.25	< 11.1%	[8]
NPC/Cu	Cu foam	No	> 15.9	< 5.7%	[9]
N,P-VG @CC	Carbon cloth	Yes	> 10.3	< 2.9%	[10]
Fe_2O_3@C@ MoS_2/CFC	Carbon cloth	Yes	> 11.5	< 13.0%	[11]
ZnNiCo-P	Ni foam	No	> 16.25	< 7.7%	[12]
CPO-27	Ni foam	No	> 17.2	< 12.8%	[13]
$NiCo_2S_4$-HNA	Ni foam	No	> 17.0	< 11.8%	[14]
Cu_3P@C	Cu foam	No	> 15.7	< 4.5%	[15]

Note: The mass of skeleton in most literatures are unavailable except in [5] and [6]. For the convenience of statistics, the minimum mass of Ni foam, Cu foam and carbon cloth are 15, 15, 10 mg cm^{-2} (it is lower than most experiments).

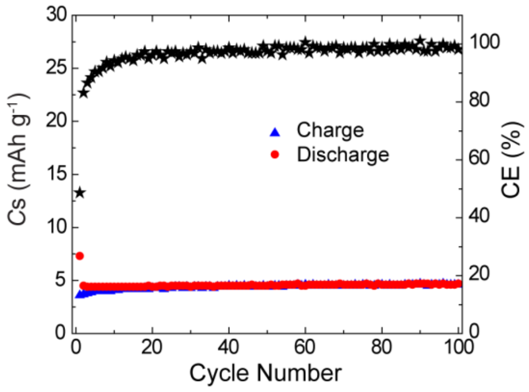

Figure S11 Cycling performance of face mask at a current density of 100 mA g^{-1}.

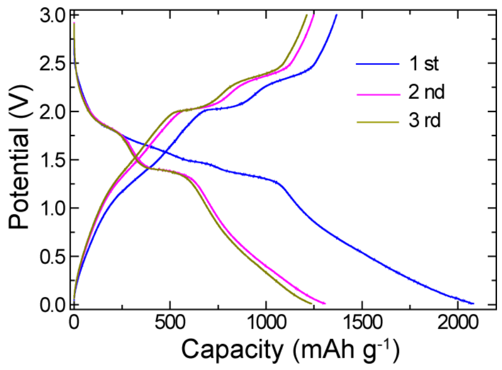

Figure S12 The discharge-charge profiles of MG@CMS/CMS@rGO for the first three cycles at a current density of 100 mA g^{-1}.

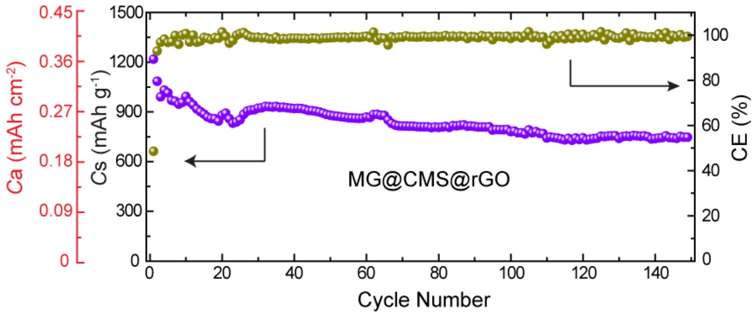

Figure S13 The cycling performance of MG@CMS@rGO at 0.1 A g^{-1}.

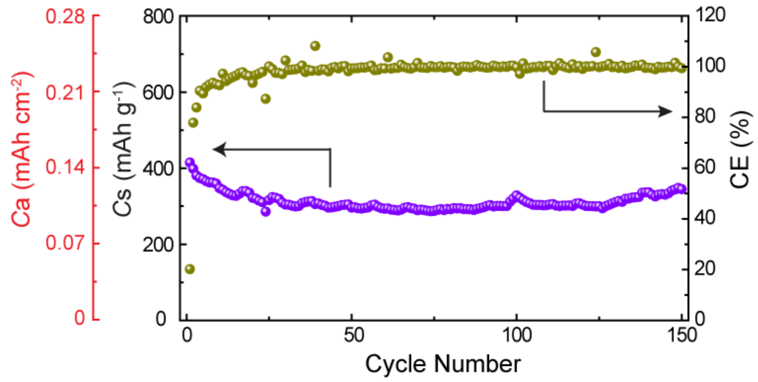

Figure S14 The cycling performance of MG@CMS/CMS@rGO without middle and outermost rGO layers at 0.1 mA g^{-1}.

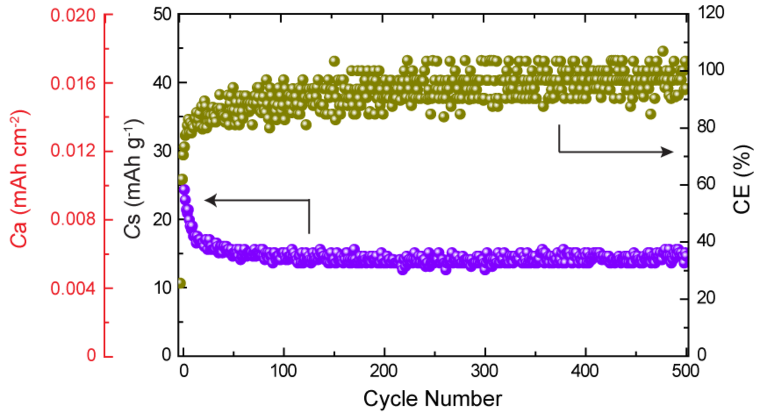

Figure S15 The cycling performance of MG@CMS/CMS@rGO without middle and outermost rGO layers at 1 A g^{-1}.

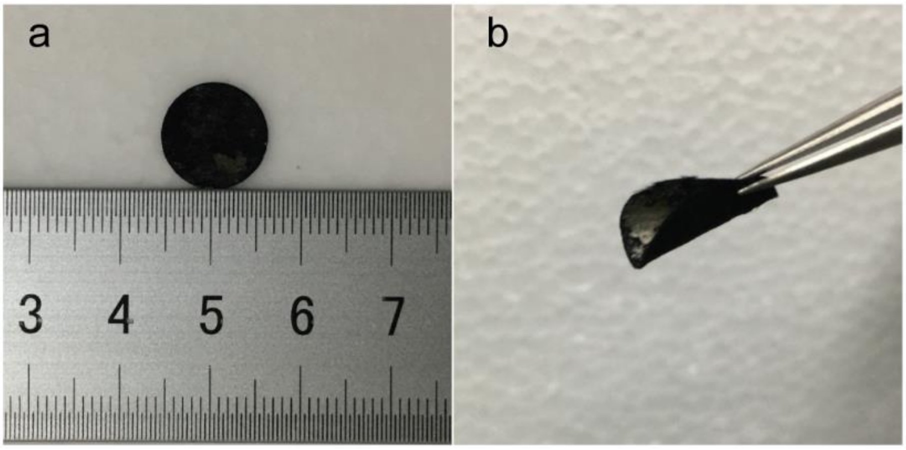

Figure S16 Digital photographs of MG@CMS/CMS@rGO after cycling test with a) integrity and b) flexibility, indicating the excellent stability for mask fibers skeleton and multilayer structure in MG@CMS/CMS@rGO.

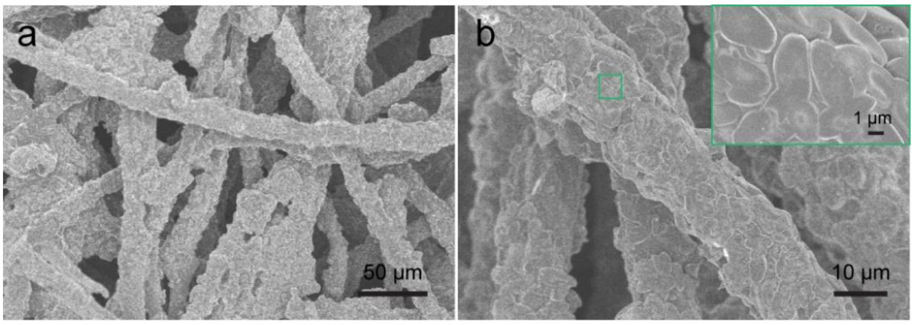

Figure S17 The SEM images of MG@CMS/CMS@rGO without middle and outermost rGO layers after 500 cycles at 1 A g^{-1}.

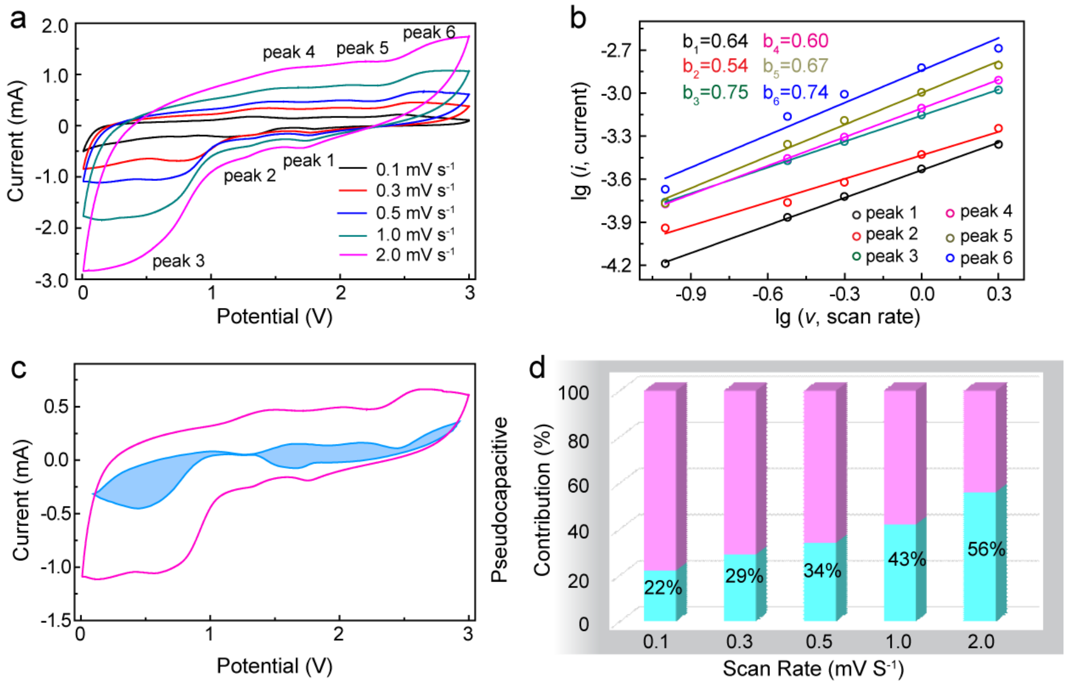

Figure S18 a) The CV curves of the MG@CMS/CMS@rGO electrode at different scan rates in one cell. b) Linear relationship of lg (*i*) and lg (*v*) at each redox peak of the MG@CMS/CMS@rGO electrode. c) Capacitive contributions separated with cyclic voltammogram at a scan rate of 0.5 mV s^{-1}. d) The capacity contributions at different scan rates.

The lg (*i*, current) versus lg (*v*, scan rate) curves (Figure S18b) obtained from the linear relationship of *i* and *v* of redox peaks as shown in the following equations 1 and 2. If the value of b is close to 1, the electrochemical reaction is dominated by pseudocapacitance; if the value of b is close to 0.5, Li^+ diffusion dominates the electrochemical process. From the fitting results, the b-values (0.64, 0.54, 0.75, 0.60, 0.67 and 0.74) imply that the system exhibits partial pseudocapacitive behavior. It is beneficial for the superior high-rate performance.

$$i = av^b \qquad (1)$$

$$\lg(i) = b\lg(v) + \lg(a) \qquad (2)$$

Where i is the peak current, ν is scan rate, a and b are adjustable parameters.

The contribution proportion of the pseudocapacitive charge storage can be calculated by below equation 3 and 4:

$$i = k_1\nu + k_2\nu^{0.5} \qquad (3)$$

$$i/\nu^{0.5} = k_1\nu^{0.5} + k_2 \qquad (4)$$

The i-value is composed of two parts: the pseudocapacitive effective ($k_1\nu$) and diffusion-controlled insertion ($k_2\nu^{0.5}$). The k_1 and k_2 are fixed for the same electrochemical reaction. Therefore, the different currents of the same voltage position at different scan rates to calculate k_1 and k_2 using the equation 4. The percentages of pseudocapacitive contribution $\left(P_{\text{pseudocapacitance}} = k_1\nu/(k_1\nu + k_2\nu^{0.5})\right)$ can be calculated by using equation 3. Take the case with a scan rate of 0.5 mV s^{-1} as an example, the total current (i, peak line) is obtained experimentally and the shadow region is the current responses of pseudocapacitive effects. Then, the percentage of pseudocapacitive contribution in a whole galvanostatic charge/discharge (GCD) processes can be obtained by integrating the whole peak line and shadow regions (Figure 18c). The proportions of pseudocapacitive charge storage increase with the increasing scan rates (22%, 29%, 34%, 43% and 56% at the scan rates of 0.1, 0.3, 0.5, 1.0 and 2.0 mV s^{-1}), resulting in the superior high-rate capability of MG@CMS/CMS@rGO electrode.

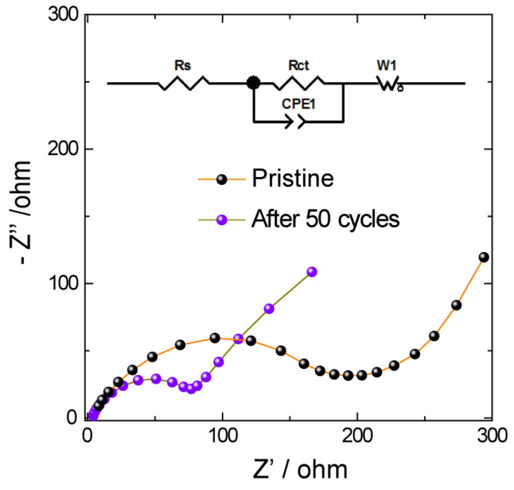

Figure S19 EIS of MG@CMS/CMS@rGO after different cycles and the inset is an equivalent electrical circuit.

Table S5 Fitting results obtained from the EIS data.

State	R_s (Ω)	R_{ct} (Ω)
Pristine	3.113	162.5
After 50 cycles	3.228	57.4

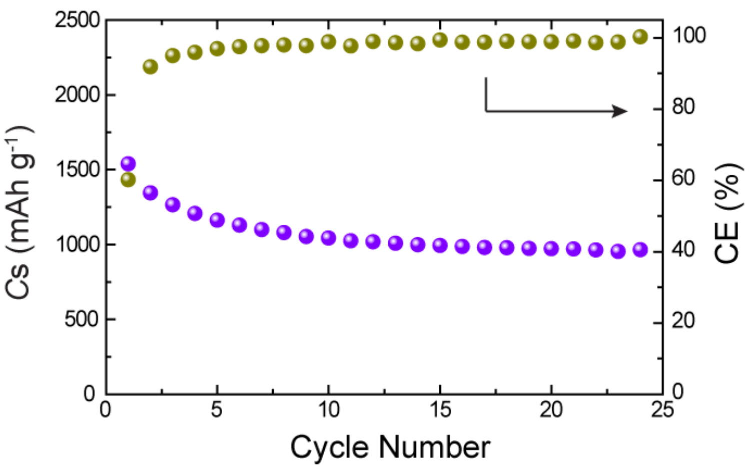

Figure S20 The cycling performance of MG@CMS/CMS@rGO at 100 mA g^{-1} at 40 °C.

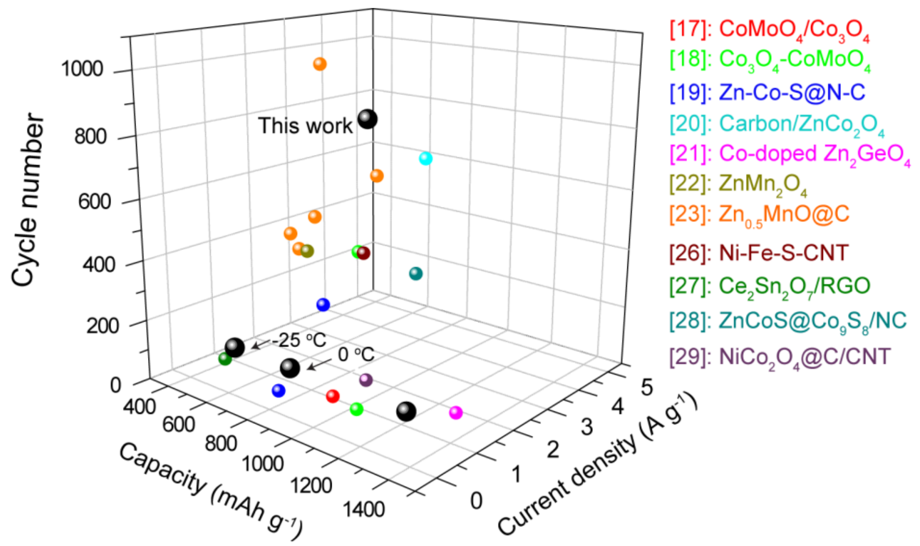

Figure S21 Comparison of long cycling performance of MG@CMS/CMS@rGO with previously reported bimetallic oxides/sulfides (just this work with excellent low temperature performance).

Table S6 Comparison of long cycling performance of MG@CMS/CMS@rGO with previously reported ternary metal oxides/sulfides.

Samples	operation temperature (°C)	Current (A g^{-1})	Reversible Capacity (mAh g^{-1})	Cycle number	Capacity retention	Ref.
**MG@CMS/CMS@rGO**	25	**0.1**	**1368**	**150**	**90**	**This work**
		**1**	**1058**	**900**	**85.2**	
$CoNi_2S_4$@PCS/CNTs	25	1	~1250	600	~64	[16]
$CoMoO_4/Co_3O_4$ hollow porous octahedrons	25	0.2	1093	100	96.1	[17]
Co_3O_4-$CoMoO_4$ HLHs	25	1	1018.2	500	~83	[18]
Zn-Co-S@N-C	25	1	860.8	300	77.6 (Compared to the second cycle capacity)	[19]
Carbon/$ZnCo_2O_4$ nanotubes	25	5	659	600	75	[20]

Co-doped Zn_2GeO_4 hollow microspheres	25	1	1419	100	62	[21]
$ZnMn_2O_4$	25	0.5	889	500	79.2	[22]
$Zn_{0.5}MnO$@C HHNDs	25	2	670	1000	~81	[23]
CoC_2O_4@CoO/Co composite	25	0.2	836.5	200	95.5 (Compared to the second cycle capacity)	[24]
$CoSeO_3$	25	3	1300	1400	56	[25]

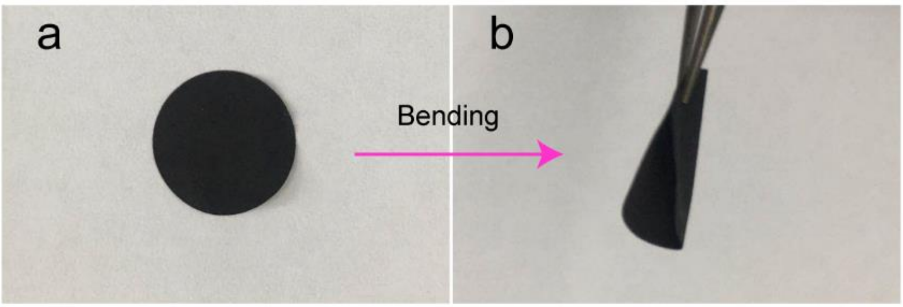

Figure S22 a) The photograph of $LiFePO_4$/SWCNT film, b) bending state, indicating the $LiFePO_4$/SWCNT cathode with desirable flexibility.

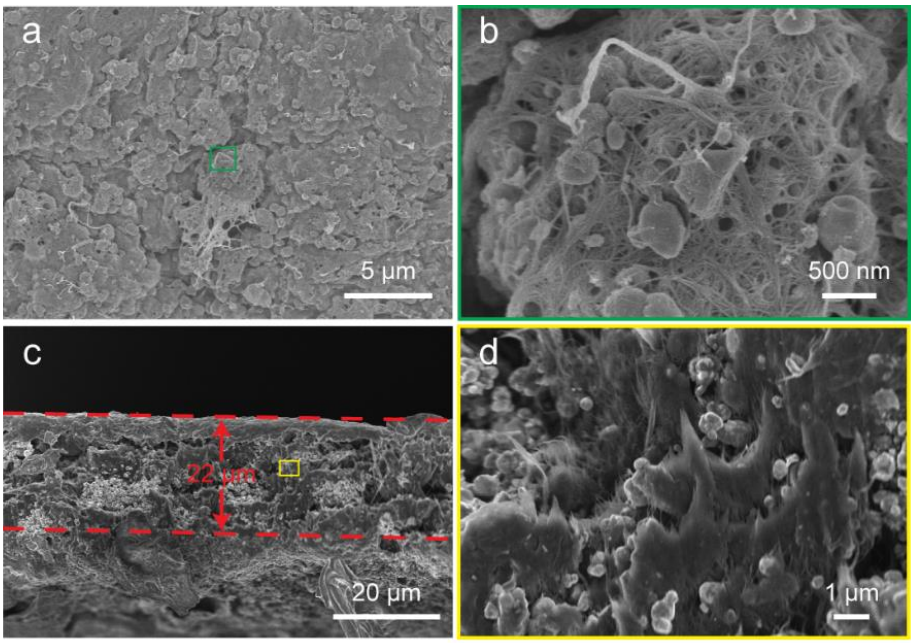

Figure S23 The SEM images of $LiFePO_4$/SWCNT film: a, b) top-view images, c, d) cross-sectional images. It is clear that the $LiFePO_4$ active materials are uniformly incorporated into SWCNT network and the thickness of $LiFePO_4$/SWCNT film is ~22 μm.

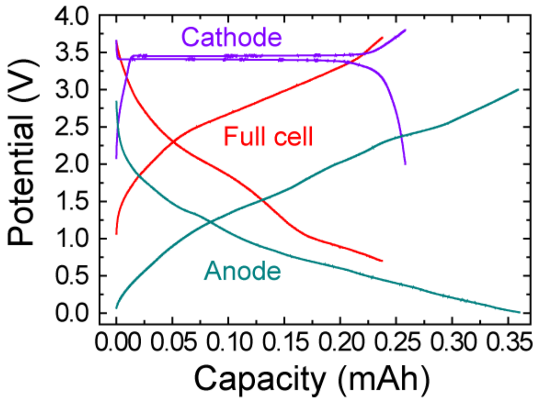

Figure S24 Charge-discharge curves of MG@CMS/CMS@rGO//$LiFePO_4$/SWCNT full cell and the correspongding $LiFePO_4$/SWCNT cathode and MG@CMS/CMS@GO anode in half cells.

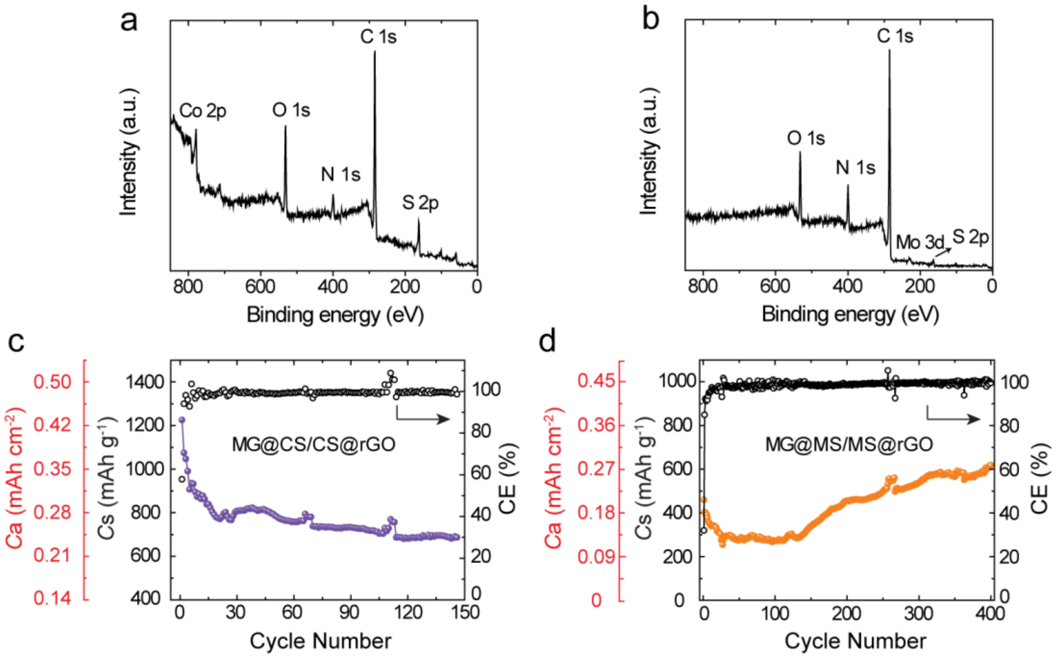

Figure S25 XPS spectra of a) MG@CS/CS@rGO and b) MG@MS/MS@rGO, just Co/Mo, S, C, N, and O exist in these two counterparts, indicating the presence of pure Co-S and Mo-S. Cycling performances of c) MG@CS/CS@rGO and d) MG@MS/MS@rGO at 100 mA g^{-1}.

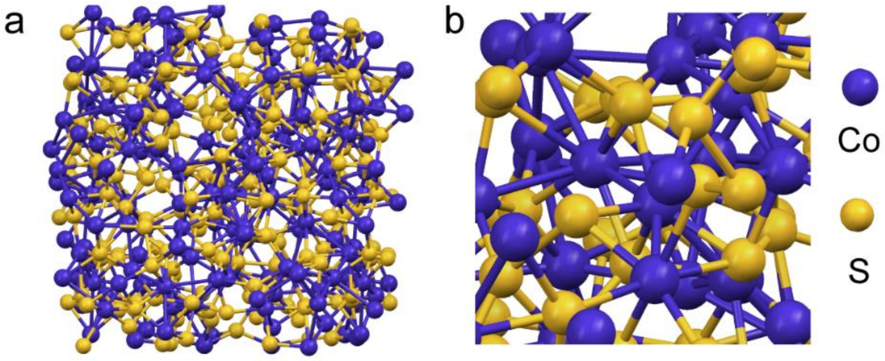

Figure S26 a) The optimized cluster structures of Co-S and b) the magnified image of (a).

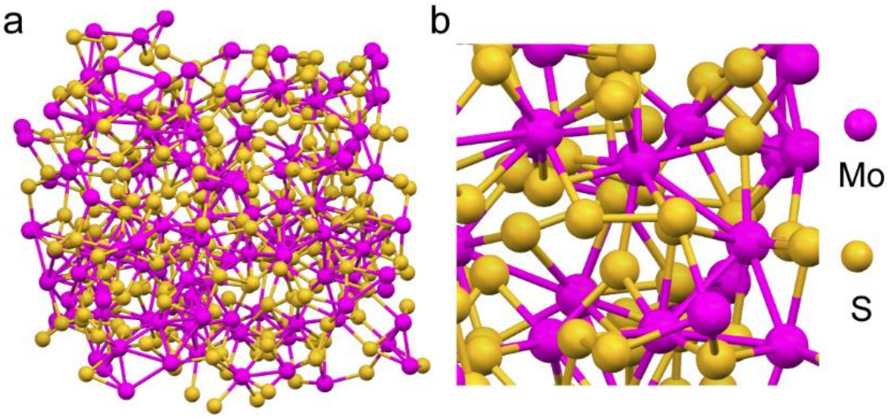

Figure S27 a) The optimized cluster structures of Mo-S and b) the magnified image of (a).

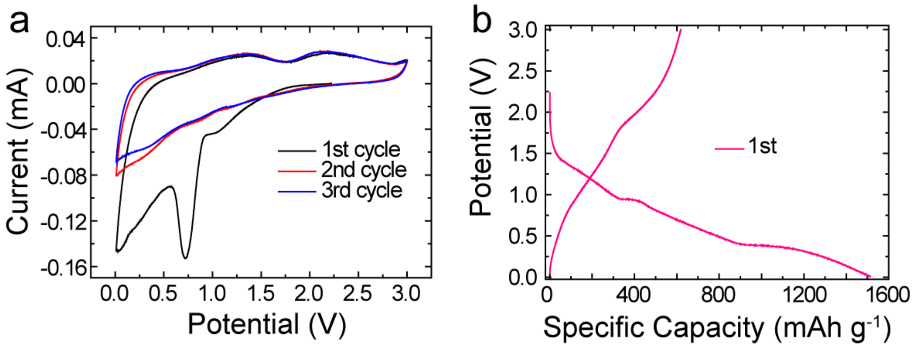

Figure S28 a) CV curves for first three cycles; b) discharge-charge profile for first cycle of MG@CMS/CMS@rGO as anode of SIBs.

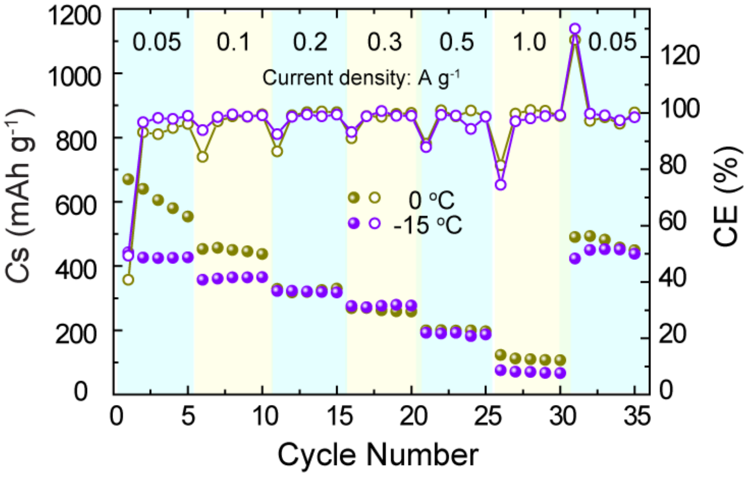

Figure S29 The rate performance of MG@CMS/CMS@rGO as SIBs anode at different current densities at 0 ^{o}C and -15 ^{o}C, respectively.

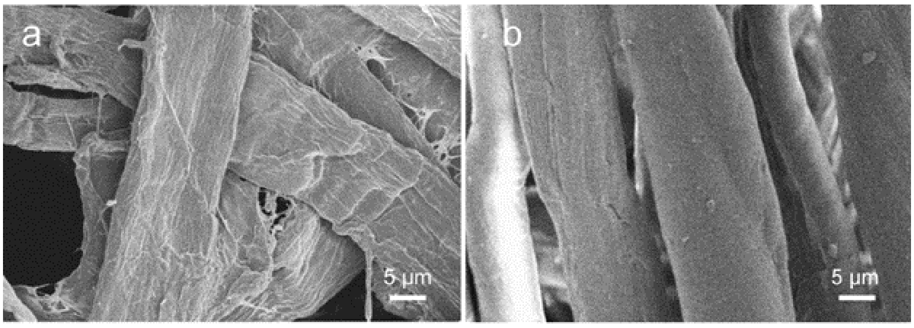

Figure S30 SEM images of pristine a) handkerchief paper and b) cloth, showing the different morphologies from the mask fibers.

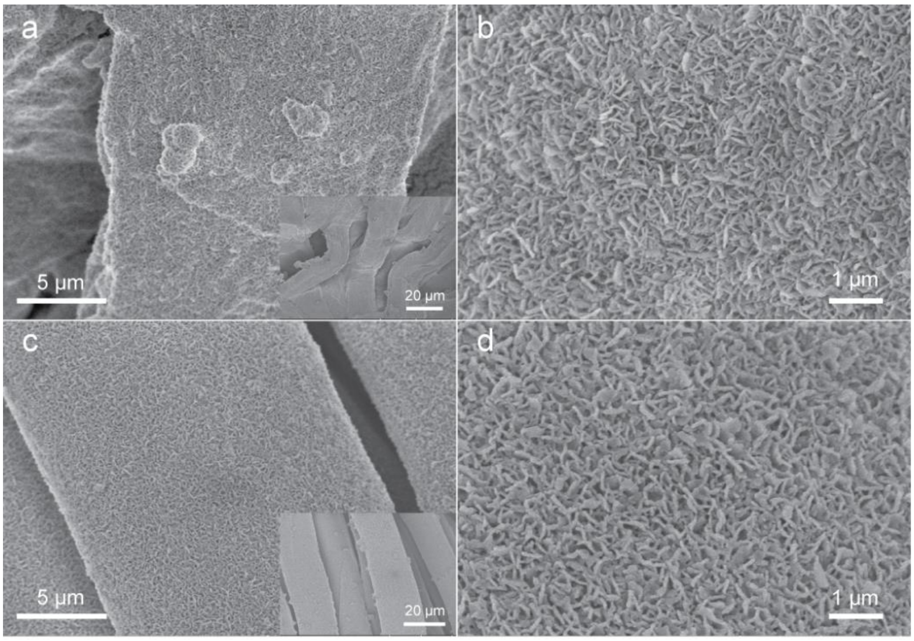

Figure S31 The SEM images of expending experiments products, adopting (a, b) handkerchief paper, (c, d) cloth as the substrates.

Commercial handkerchief paper and cloth with good hydrophilicity were adopted to replace face mask as skeletons. Although, the primary morphologies of handkerchief paper and cloth are different from the face mask (Figure S30), the same structure as MG@CMS/MS@rGO is obtained through the same procedures.

References

[1] W. Ai, W. Zhou, Z. Du, Y. Du, H. Zhang, X. Jia, L. Xie, M. Yi, T. Yu, W. Huang, *J. Mater. Chem.* **2012**, *22*, 23439-23446.

[2] J. Ni, X. Zhu, Y. Yuan, Z. Wang, Y. Li, L. Ma, A. Dai, M. Li, T. Wu, R. Shahbazian-Yassar, J. Lu, L. Li, *Nat. Commun.* **2020**, *11*, 1212.

[3] J. Zhang, K. Zhang, J. Yang, G.-H. Lee, J. Shin, V. Wing-hei Lau, Y.-M. Kang, *Adv. Energy Mater.* **2018**, *8*, 1800283.

[4] J.-M. Son, S. Oh, S.-H. Bae, S. Nam, I.-K. Oh, *Adv. Energy Mater.* **2019**, *9*, 1900477.

[5] B. Wang, J. Ryu, S. Choi, X. Zhang, D. Pribat, X. Li, L. Zhi, S. Park, R. S. Ruoff, *ACS Nano* **2019**, *13*, 2307-2315.

[6] M. Ramu, J. R. Chellan, N. Goli, P. Joaquim, V. Cristobal, B. C. Kim, *Adv. Funct. Mater.* **2020**, *30*, 1906586.

[7] J. Balamurugan, C. Li, V. Aravindan, N. H. Kim, J. H. Lee, *Adv. Funct. Mater.* **2018**, *28*, 1803287.

[8] M. S. Javed, H. Lei, H. U. Shah, S. Asim, R. Raza, W. Mai, *J. Mater. Chem. A* **2019**, *7*, 24543-24556.

[9] Y. An, Y. Tian, Y. Li, S. Xiong, G. Zhao, J. Feng, Y. Qian, *J. Mater. Chem. A* **2019**, *7*, 21966-21975.

[10] W. Qiu, H. Xiao, Y. Li, X. Lu, Y. Tong, *Small* **2019**, *15*, 1901285.

[11] J. Zhan, K. Wu, X. Yu, M. Yang, X. Cao, B. Lei, D. Pan, H. Jiang, M. Wu, *Small* **2019**, *15*, 1901083.

[12] J. Li, Z. Liu, Q. Zhang, Y. Cheng, B. Zhao, S. Dai, H.-H. Wu, K. Zhang, D. Ding, Y. Wu, M. Liu, M.-S. Wang, *Nano Energy* **2019**, *57*, 22-33.

[13] D. Zhou, J. Ni, L. Li, *Nano Energy* **2019**, *57*, 711-717.

[14] Y. Cui, J. Zhang, C. Jin, Y. Liu, W. Luo, W. Zheng, *Small* **2019**, *15*, 1804318.

[15] S. Ni, B. Zheng, J. Liu, D. Chao, X. Yang, Z. Shen, J. Zhao, *J. Mater. Chem. A* **2018**, *6*,

18821-18826.

[16] Z. Jia, Z. Cui, Y. Tan, Z. Liu, X. Guo, *Chem. Eng. J.* **2019**, *370*, 89-97.

[17] Y. Chen, Y. Wang, X. Shen, R. Cai, H. Yang, K. Xu, A. Yuan, Z. Ji, *J. Mater. Chem. A* **2018**, *6*, 1048-1056.

[18] C. He, B. Han, S. Han, Q. Xu, Z. Liang, J. Y. Xu, M. Ye, X. Liu, J. Xu, *J. Mater. Chem. A* **2019**, *7*, 26884-26892.

[19] X. Wei, Y. Zhang, B. Zhang, Z. Lin, X. Wang, P. Hu, S. Li, X. Tan, X. Cai, W. Yang, L. Mai, *Nano Energy* **2019**, *64*, 103899.

[20] H. Li, L. Lv, W. Wang, X. Huang, D. Chen, *J. Mater. Chem. A* **2019**, *7*, 22642-22649.

[21] J. Lu, D. Li, L. Li, Y. Chai, M. Li, S. Yang, J. Liang, *J. Mater. Chem. A* **2018**, *6*, 5926-5934.

[22] Z. Zhao, G. Tian, A. Sarapulova, V. Trouillet, Q. Fu, U. Geckle, H. Ehrenberg, S. Dsoke, *J. Mater. Chem. A* **2018**, *6*, 19381-19392.

[23] D. Wang, W. Zhou, R. Zhang, X. Huang, J. Zeng, Y. Mao, C. Ding, J. Zhang, J. Liu, G. Wen, *J. Mater. Chem. A* **2018**, *6*, 2974-2983.

[24] Z. He, L.-a. Huang, J. Guo, S.-e. Pei, H. Shao, J. Wang, *Energy Storage Mater.* **2020**, *24*, 362-372.

[25] G. D. Park, J. H. Hong, J. H. Choi, J.-H. Lee, Y. S. Kim, Y. C. Kang, *Small* **2019**, *15*, 1901320.

[26] S. Zhang, G. Wang, B. Wang, J. Wang, J. Bai, H. Wang, *Adv. Funct. Mater.* **2020**, *30*, 2001592.

[27] Q. Wu, Y. Liu, H.-g. Wang, J. Hou, Y. Li, Q. Duan, *J. Mater. Chem. A* **2020**, *8*, 5517-5524.

[28] M. K. Aslam, S. S. A. Shah, S. Li, C. Chen, *J. Mater. Chem. A* **2018**, *6*, 14083-14090.

[29] H. Wang, L. Hu, C. Wang, Q. Sun, H. Li, T. Zhai, *J. Mater. Chem. A* **2019**, *7*, 3632-3641.
